# Gram‐Negative Bacteria Across Spatial Scales: A Meta‐Analysis of Ant‐Associated Bacterial Communities Under Distinct Environmental Conditions

**DOI:** 10.1002/ece3.72425

**Published:** 2025-10-30

**Authors:** M. R. Bitar, M. Azevedo‐Silva, P. S. Oliveira, G. Q. Romero, S. P. Ribeiro

**Affiliations:** ^1^ Laboratório de Ecologia do Adoecimento & Florestas NUPEB/ICEB Universidade Federal de Ouro Preto Ouro Preto Brazil; ^2^ Departamento de Biologia Animal Universidade Estadual de Campinas (UNICAMP) Campinas Brazil; ^3^ Department of Ecology and Evolutionary Biology University of Michigan Ann Arbor Michigan USA

**Keywords:** 16S rRNA, ant‐associated bacterial communities, environmental variability, gram‐negative bacteria, host resistance, meta‐analysis, social insects

## Abstract

Shaped by ecological and evolutionary factors, microbial communities influence host health and resistance to environmental stressors. Ants that host diverse bacterial communities may rely on these communities for adaptation to different environmental conditions. This meta‐analysis investigates the proportion of Gram‐negative (GN) bacteria in ants' bacterial communities (gut and whole body) under contrasting environments at distinct scales: (i) arboreal vs. ground habitats and (ii) tropical vs. temperate zones. We hypothesize that ants under greater environmental variability (arboreal and in temperate zones) host higher proportions of GN bacteria, which are better suited to extreme ecological pressures. We analyzed data from 193 ant bacterial communities across 27 studies and found that ants from temperate regions and arboreal microhabitats do harbor higher proportions of GN bacteria compared to those from tropical regions or ground microhabitats. This suggests that GN bacteria may confer adaptive advantages in variable environments, potentially enhancing host resistance to stressors. Our findings underscore the role of abiotic ecological factors in shaping ant‐associated bacterial communities and highlight the need for further research on how GN bacteria contribute to insect survival in less stable environments. Future studies should explore the functional roles of GN bacteria in host resistance, particularly regarding climate change and ecosystem disruptions.

## Graphical abstract

Using a meta‐analysis, we estimated the proportion of Gram‐negative (GN) bacteria in ant microbiota across different environments, focusing on habitat and climate influences. Our findings show that ants from temperate regions and arboreal habitats harbor higher proportions of GN bacteria, potentially offering adaptive advantages in variable environments. This advantage may stem from GN bacteria's outer membrane, which provides protection against antibiotics and aids in antimicrobial enzyme production, enhancing microbial resilience in fluctuating ecological conditions.

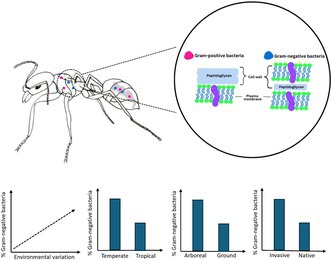

## 1 Introduction

Bacterial communities play a crucial role in insect behavior, ecology, and evolution (Mondal et al. 2023; Zhang, Zhang, et al. 2023). The interaction between hosts and their beneficial bacteria can influence insect survival under varying environmental conditions (Gupta and Nair 2020). Gut bacteria, for example, provide metabolic pathways adapted to specific ecological niches, helping insects with nutrition, development, and pathogen defense (Chen et al. 2016). The composition of an insect's bacterial communities can be diverse and species‐specific, shaped by geographic factors such as latitude, altitude, and local environmental conditions like temperature and soil properties (Lange et al. 2023; Magoga et al. 2023). These factors influence the bacterial assemblages to which insects are exposed, thus impacting the structure of their own microbial communities (Hannula et al. 2019; Harvey et al. 2023). For instance, environmental factors such as temperature and precipitation have been linked to symbiont abundance and insecticide resistance in the brown planthopper ( *Nilaparvata lugens* ), indicating that climate not only shapes bacterial composition but also affects host‐environment interactions (Zhang, Cai, et al. 2023). Additionally, these communities play a crucial role in regulating energy homeostasis under extreme temperatures, underscoring the potential for coevolution between hosts and their bacteria (Khakisahneh et al. 2020; Raza et al. 2020; Wang et al. 2024).

Ants, one of the most diverse and ecologically dominant insect groups (Hölldobler and Wilson 1990), have established successful evolutionary trajectories partly through interactions with microorganisms (Boursaux‐Eude and Gross 2000; Moreau 2020). These symbiotic relationships have contributed to ants' ecological success; for instance, some arboreal ants depend on associated microorganisms to thrive on phloem‐derived diets, which are limited in essential nutrients (Pringle and Moreau 2017; Russell 2017). However, not all members of the microbiome are beneficial. Microbial associates can also include commensals, which neither harm nor benefit the host, and pathogens, which may negatively impact ant health (Hammer et al. 2019; Moreau 2020). Despite these varied roles, the presence of all microbial members contributes to the overall balance, structure, and stability of the host's bacterial communities, influencing community dynamics and host–microbe interactions (Coyte et al. 2015; Engel and Moran 2013). Ant‐associated bacterial communities are influenced by a variety of factors, including diet (Hu et al. 2014), social interactions (Ivens et al. 2018), environmental conditions (Lucas et al. 2017), colony structure (Green and Klassen 2022), invasiveness (Hu et al. 2017), pathogen pressure (Sapountzis et al. 2019), and genetics (Segers et al. 2019). The diversity and composition of ants' bacterial communities provide valuable insights into ant biology and ecology (Lucas et al. 2019; Rocha et al. 2023; Ronque et al. 2020). Understanding these microbial communities is crucial to understanding how they contribute to the ecological success of ants, particularly under distinct environmental conditions (Hölldobler and Wilson 1990).

Gram‐negative and Gram‐positive bacteria are two major groups of bacteria distinguished by their cell wall structure and gram‐staining characteristics, offering valuable insights into microbial diversity and how it is shaped by environmental factors due to their contrasting tolerances and ecological strategies (Cao et al. 2021; Fanin et al. 2019). Gram‐positive bacteria have a thick peptidoglycan layer that retains the crystal violet stain, whereas Gram‐negative bacteria possess a thinner peptidoglycan layer and an outer membrane containing lipopolysaccharides (LPS), which confer protection against antibiotics and facilitate the production of antimicrobial enzymes (Gupta 2011; Silhavy et al. 2010). Gram‐negative bacteria also release outer membrane vesicles (OMVs) that sequester antimicrobial peptides, reducing their efficacy and further enhancing survival under strong selective pressures (Balhuizen et al. 2021). This resistance may give Gram‐negative bacteria a competitive advantage over other bacteria in variable environments with fluctuating ecological conditions (Atanaskovic et al. 2022; Nikaido 2003; Schwechheimer et al. 2013). Additionally, many Gram‐negative bacteria have larger genomes than Gram‐positive bacteria, and consequently more metabolic flexibility, an important adaptive trait (Konstantinidis and Tiedje 2005). Although less explored, such benefits may extend to the hosts, also modulating their responses to external conditions.

In insects, several Gram‐negative symbionts from the genera *Buchnera*, *Hamiltonella*, *Pantoea*, *Morganella*, *Stammera*, and *Wolbachia* contribute to host functions such as nutrition, defense, detoxification, communication, digestion and reproductive manipulation (Kaltenpoth et al. 2025). Some studies highlight the beneficial role of Gram‐negative bacteria in modulating host physiological responses (Montllor et al. 2002; Moran et al. 2005), such as the presence of a facultative Proteobacteria endosymbiont that enhances aphid resistance to heat stress (Montllor et al. 2002). Temperature changes are also shown to alter the abundance and composition of ant‐associated bacteria (McMunn et al. 2022). In this context, ants under less stable environments (characterized, for example, by higher temperature variation; Vinod et al. 2023) can benefit from the presence of Gram‐negative bacteria for responding to these challenging environments. Notably, the arboreal ant species *Azteca chartifex* was found to associate predominantly with Gram‐negative bacteria, commonly from the genus *Enterobacter*, which may protect against contamination by environmental Gram‐positive bacteria, potentially facilitating successful colonization of the tree canopy, a habitat that may experience higher environmental variability (Bitar et al. 2021). Despite the ecological importance of these findings, the assessment of bacterial community composition by quantifying the proportion of Gram‐positive (GP) versus Gram‐negative (GN) bacteria has hardly been explored, and no study has examined how these groups associate with ants' bacterial communities across distinct environmental conditions. Although GN and GP do not represent monophyletic bacterial clades, they capture fundamental differences in cell wall architecture that are linked to ecological and functional traits, such as resistance to environmental stressors and interactions with hosts. From an ecological perspective, quantifying these groups provides a useful proxy to investigate how bacterial community structure may influence host adaptation to less stable or challenging environments (Galdiero et al. 2012).

In this study, we analyze the GN bacteria proportion in ants' bacterial communities (from both the gut and whole body) and explore its potential association with distinct environmental conditions at both macro (tropical vs. temperate zones) and microscale (arboreal and ground habitats). Our meta‐analysis incorporates data from 27 published reports, including 193 microbiome datasets. We define environmental variability as fluctuations in abiotic factors that may influence microbial community composition. At the macroscale, this includes seasonal variation in temperature and precipitation (e.g., amplitude of temperature between the coldest and hottest months; variation in monthly precipitation; length of dry vs. rainy seasons), with temperate habitats exhibiting greater seasonal fluctuations than tropical habitats (Lisovski et al. 2017; Liukkonen et al. 2024). At the microscale, environmental variability refers to variation in microclimatic conditions such as temperature, humidity, and light within different vertical strata of the habitat. Canopy microclimates are generally more variable than ground‐level environments due to greater exposure to sunlight, wind, and precipitation (Deng et al. 2022; Leahy et al. 2021; Scheffers and Williams 2018).

**FIGURE 1:**
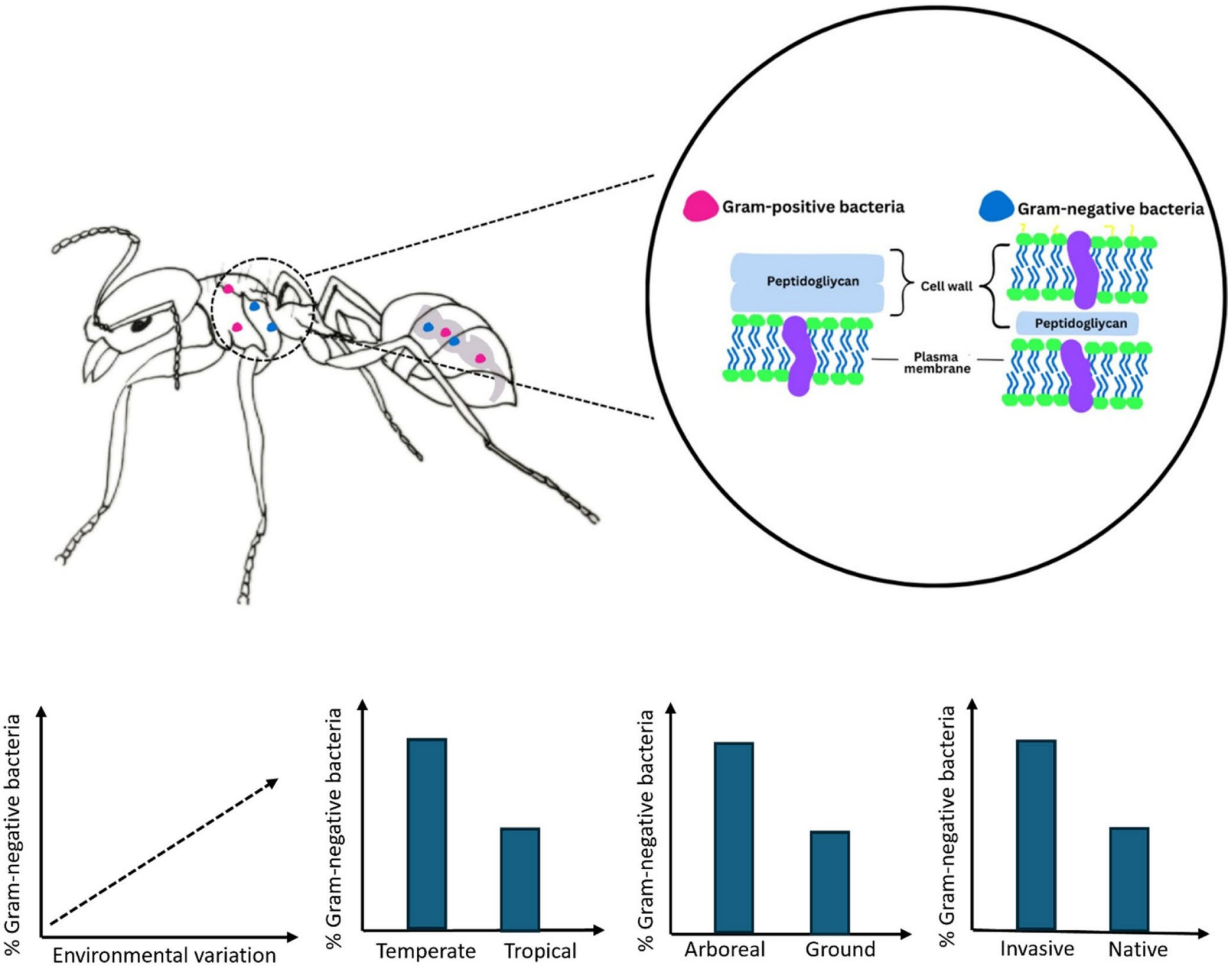
Scheme showing ants' bacterial communities (from gut and whole body) and the difference in membrane composition of Gram‐positive and Gram‐negative bacteria. Graphs of the hypothesis tested in the present study are also shown. Figure created by the author using Canva (www.canva.com).

Given that GN bacteria are more likely to thrive in less stable environments, we hypothesize that the proportion of GN bacteria will be higher in environments with greater environmental variability. Accordingly, we predict that: (1) GN bacteria will be more abundant in ants from temperate habitats than in those from tropical habitats, as a result of the greater seasonal variation in temperate zones; (2) GN bacteria will be more prevalent in the bacterial communities of canopy‐dwelling ants compared to ground‐dwelling ants, due to the greater microclimatic variability in the canopy; (3) Additionally, invasive ants will host higher proportions of GN bacteria than native ants, as invasives frequently encounter novel environmental conditions (Marsico et al. 2010) (Figure 1). Our study is the first to demonstrate that the proportion of Gram‐negative bacteria in ants' bacterial communities is linked to environmental variability at both macro and microscale, offering new insights into how bacterial community structure can influence the ecological success and dominance of ants in terrestrial ecosystems.

## 2 Materials and Methods

### 2.1 Literature Review—Identification and Screening

**FIGURE 2:**
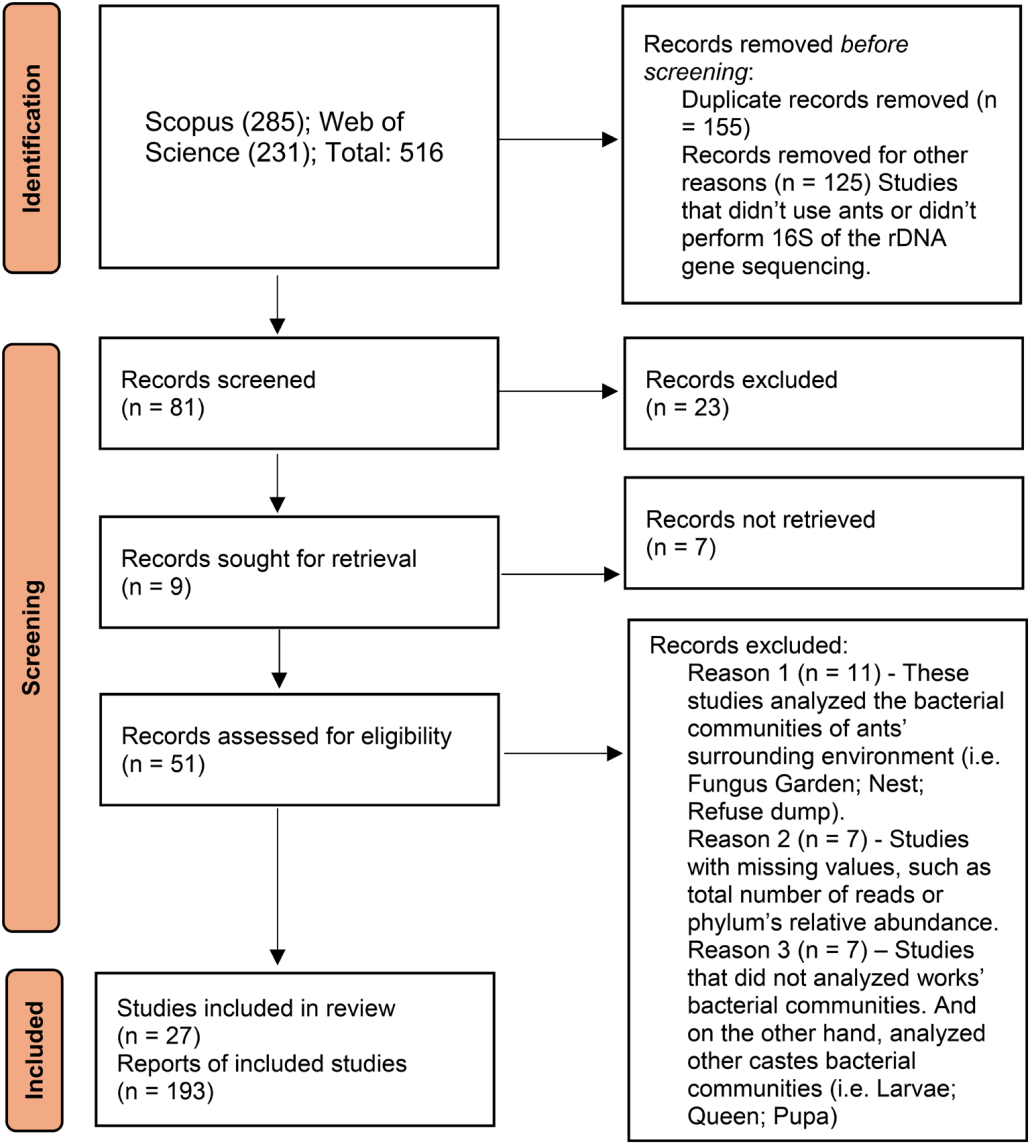
Preferred Reporting Items for Systematic Reviews and Meta‐Analyses (PRISMA) filtration of journal articles.

To assess the proportion of Gram‐positive and Gram‐negative bacteria in ants' bacterial communities, a comprehensive search was conducted for peer‐reviewed studies analyzing ant‐associated bacteria. Literature was searched in Elsevier's *Scopus* and Clarivate Analytics' *Web of Science* (title, abstract and keywords = (ant AND 16S*) OR (ant AND bacterial communities*)) for articles published between 2000 and 2021, a period corresponding to the widespread use of molecular methods, including 16S rRNA sequencing, to characterize ant‐associated bacterial communities. Only articles published in English were included. Initial searches yielded a total of 231 papers from Web of Science and 285 papers from Scopus. Exclusion criteria were applied to filter out studies not directly relevant to the current review. Articles without ants or lacking 16S rRNA amplicon sequencing, as well as those focusing only on the bacteria of ants' surrounding environments (nest, fungus garden, dump, nearby soil, or employing culture‐dependent methods) were excluded. Additionally, studies investigating specific symbionts or lacking essential data such as total sequence numbers and relative abundance of bacterial phylum were also excluded (Figure 2). After the screening process, a total of 27 studies met all inclusion requirements and were retained for the upcoming analyses. Because a single study can contain more than one bacterial community information, from the 27 studies we analyzed 193 ant bacterial communities' data outputs, of which 187 identified ants at species (representing 51 ant species across 28 genera) and 6 studies only reported ants at genus level (Table S1). Each output represents the bacterial community of an ant colony, with some colonies defined by collection location. Because the original studies differed in data organization, information was extracted carefully to represent each colony as accurately as possible. This study followed the instructions of the Preferred Reporting Items for Systematic Reviews and Meta‐Analyses (Page et al. 2021) (Figure 2).

### 2.2 Data Collection

From each bacterial community's data output (*N* = 193), we collected data on ant host species (or genus), caste, bacterial community type, ant microhabitat (ground or arboreal), diet, geographic region, and total number of reads. Bacterial community type was classified as either whole ant body (where total DNA was extracted from the external body) or ant gut (where total DNA was extracted from the digestive tract). When diet or habitat information was not directly reported in the original studies, these traits were retrieved from complementary sources based on the ant species name (e.g., additional literature and ant public databases). The final dataset was also revised by an expert to confirm the accuracy of these classifications. Environmental variation was defined primarily in terms of climate. To assign climate categories, the geographic location (or geographic coordinates, when available) of each ant sample was first extracted and categorized into five biogeographic regions following Wallace's (1876) classification. After this initial step, the samples were grouped into two broader geographic regions, tropical and temperate, to address the research question. Bacteria were categorized as Gram‐positive, Gram‐negative, or from the phylum Tenericutes. Acid‐fast bacteria, such as *Mycobacterium*, were treated as Gram‐positive in our analyses, although they possess a waxy mycolic acid layer rather than the conventional peptidoglycan cell wall. Some ant species are also associated in abundance with bacteria from the class *Mollicutes* (Tenericutes), which lack a cell wall; when present, their proportion was also recorded. The proportions of Gram‐positive (GP) and Gram‐negative (GN) bacteria were determined based on the relative abundance of each phylum within each sample. Phyla with abundance less than 1% were classified as “others”. When available, this information was obtained using the number of reads; otherwise, information was extracted from relative abundance bar plots using ImageJ software (Schneider et al. 2012).

A hierarchical key scheme was constructed to assess the distribution of the studies across distinct categories (Figure S1). The studies were classified for: Bacterial community (whole ant body or ant gut), macroscale environment (temperate or tropical), invasiveness (native or invasive), microscale environment (arboreal or ground), and diet (omnivorous, herbivore, predator or fungivore). However, since ant diet is associated with microhabitat and macroscale environment, this category was excluded from the analyses but discussed based on ecological traits of each guild. For instance, herbivorous ants were all arboreal while fungivores and predatory ants were primarily ground‐dwelling in neotropical habitats (Figure S2).

### 2.3 Statistical Analysis

Whole body and gut bacterial communities were analyzed separately. The proportion of GN bacteria was analyzed in response to macroscale and microscale environments (climate and microhabitat, respectively) and invasiveness. However, given the limited number of records of whole‐body bacterial communities in arboreal ants from the tropics (Figure S1), microhabitat analyses were performed exclusively for gut bacterial communities. Similarly, due to the low number of observations of gut bacterial communities in invasive species, invasiveness was used as a predictor only for whole body bacterial communities (Figure S1). Because sample sizes varied across groups, we used generalized linear mixed models (GLMMs) with a binomial distribution, which are appropriate for unbalanced designs and account for random effects such as study identity and species. We used the glmmTMB package (Brooks et al. 2017), which accounts for zero‐inflated data. We evaluated both additive models (climate + microhabitat and climate + invasiveness for gut and whole‐body bacterial communities, respectively) and models with a single predictor. Among studies, the methodologies varied in protocols and materials for bacterial community extraction and sequencing. Therefore, we also used GLMM to evaluate if the sequencing platforms and the use of negative controls (necessary to filter out contaminant bacteria) influenced the proportion of Gram‐negative bacteria overall (i.e., whole body and gut bacterial communities were analyzed together; see Supporting Information 1). Since each author used a different extraction kit, the influence of extraction kits was also accounted for as a random effect within each study. For all models, studies were included as random effects to account for variation among them due to differences in methodology, population, or other study‐specific factors. To account for the phylogenetic non‐independence of ant species hosts, we used the most recent phylogeny published for ants (Borowiec et al. 2025). The consensus tree was pruned to include only the ant genera present in our dataset using the package ape (Paradis and Schliep 2019). Ant species were added to the pruned tree as polytomies within genus using the package phytools (Revell 2024). Due to the structure of our data and sample size, phylogenetic generalized linear mixed models (PGLMM) accounting for phylogenetic covariation failed to converge. Thus, we opted to also include species as a random effect in all GLMM models. We acknowledge that this is not the most adequate control for shared evolutionary history among ant hosts, but given methodological constraints, this was the feasible approach for accounting for ant species identity. We, however, plotted the pruned ant tree alongside the estimated proportion of GN for visualization using the package ggtree (Yu et al. 2017) and tested it for phylogenetic signal using Pagel's *λ* with the package phytools. Phylogenetic signals were tested for all bacterial communities together and for gut and whole and bacterial communities separately. For these analyses, we used the mean proportion of GN for species with more than one record. Analysis of variance (ANOVA) was used to test significant effects in all models. Finally, we also evaluated the mean groups of gram‐negative bacteria when models were significant.

We used ggplot2 and sciplot packages (Wickham 2009; Morales 2022) to construct the plots showing the percentage of GN bacteria influenced by habitat and climate (for gut bacterial community), and invasiveness and climate (for whole body bacterial communities). The graphs of habitat and climate effects on GN proportion were also created to facilitate the visualization of macro and microscale effects. All statistical analyses were made using R software (version 4.3.0) (R Core Team 2021).

## 3 Results

**FIGURE 3:**
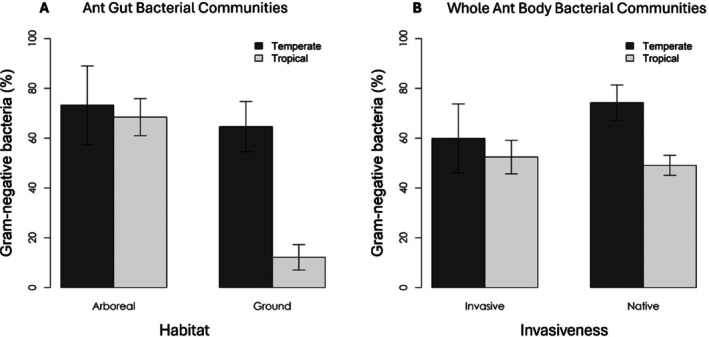
Proportion of Gram‐negative bacteria in ant's gut bacterial communities from different microscale habitats and macroscale environments. (A) Arboreal versus ground microhabitats from temperate versus tropical environments. (B) Invasive versus native ants from temperate versus tropical environments.

**FIGURE 4:**
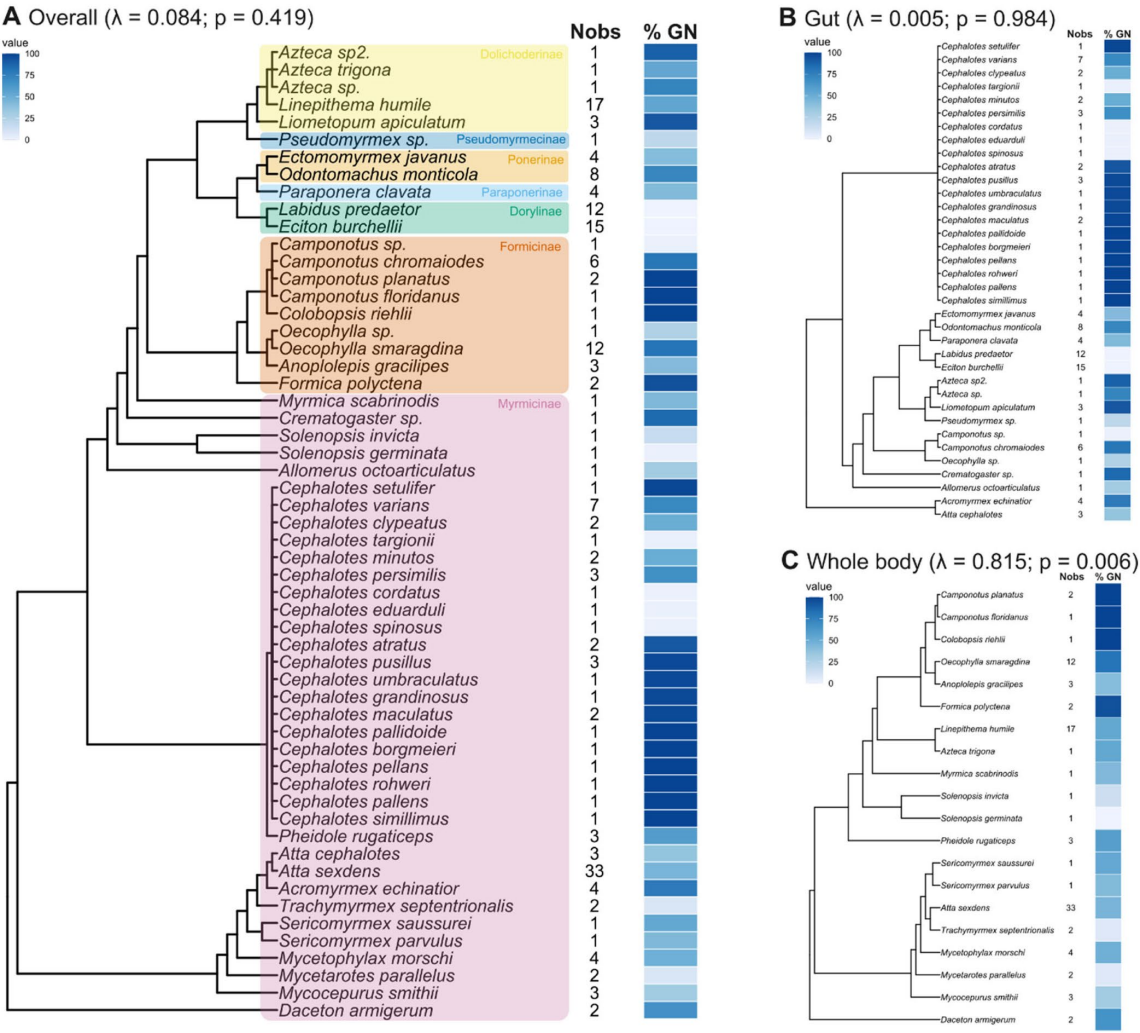
(A) Phylogeny of ant species included in this study and gram‐negative bacterial distribution among host ant species. (A) Pruned phylogenetic tree with ant subfamilies highlighted (species were added as polytomy within respective genera). Proportion of Gram‐negative (% GN) bacteria and number of observations per host ant species for (A)—overall (gut and whole body), (B)—gut, and (C)—whole body bacterial communities. For species with more than one record, mean % of GN is shown. Pagel's *λ* phylogenetic signal and the corresponding *p*‐value (*p*) are shown for each bacterial community.

We analyzed bacterial communities from 193 ant samples, representing 51 ant species across 28 ant genera and 7 subfamilies over the 27 included studies (Figure 4A; Supporting Information 2 and Table S1). Overall, when considering gut and whole‐body bacterial communities together, we found no significant phylogenetic signal for the proportion of Gram‐negative bacteria among ant hosts (Figure 4A). At both macro and microscale, we found support for our initial hypotheses. Additive model for gut bacterial communities showed strong differences across microhabitats, with arboreal ants harboring significantly higher proportions of Gram‐negative bacteria than ground‐dwelling ants (*χ* ^2^ = 7.43, *p* = 0.006; Table 1; Figure 3A) for both temperate and tropical regions (tropical: *N* = 32; temperate: *N* = 8). Climate effects, however, were weaker and not statistically significant (*χ* ^2^ = 2.46, *p* = 0.117; Table 1). In this model, species‐level differences explained substantial variance (Table 1), whereas study effects were negligible. Moreover, we found no significant phylogenetic signal for the proportion of GN in gut bacterial communities (Figure 4B), suggesting that ant identity has an important effect on shaping ant gut bacterial communities, but not necessarily their shared evolutionary history (Figure 4B). Among the higher proportions of GN bacteria in arboreal ants' gut bacterial communities, the most abundant taxa belonged to the family *Acetobacteraceae* and to the orders *Opitutales* and *Burkholderiales* (Table S2).

**TABLE 1:** Analysis of variance (ANOVA) results conducted for each model to evaluate the influence of climate, microhabitat, and ant invasiveness on the proportion of Gram‐negative bacteria in ant gut and whole‐body bacterial communities.

Model	Predictors	Chisq	Df	*p*	Random effects	Variance ± SD (random effect)
*Gut*						
Microhabitat + Climate	Microhabitat	7.43	1	0.006*	Study	0.00007 ± 0.009
	Climate	2.46	1	0.117	Species	14.21 ± 3.77
Microhabitat	Microhabitat	2.31	1	0.129	Study	2.76 ± 1.66
					Species	13.67 ± 3.7
Climate	Climate	1.48	1	0.224	Study	7 ± 2.65
					Species	12.34 ± 3.51
*Whole body*						
Invasiveness + Climate	Invasiveness	5889.2	1	< 0.001*	Study	8.17 ± 2.86
	Climate	2515.8	1	< 0.001*	Species	9.07 ± 3.01
Invasiveness	Invasiveness	10,029	1	< 0.001*	Study	7.52 ± 2.74
					Species	8.02 ± 2.83
Climate	Climate	6704.1	1	< 0.001*	Study	7.36 ± 2.71
					Species	7.65 ± 2.77

*Note:* Random effects variance and standard deviation (sd) of each GLMM are also shown.

Abbreviations: Chisq, chi‐squared statistic; Df, degrees of freedom; *p*, *p*‐value.

* Significant *p*‐values (*p* < 0.05).

Contrary to our initial expectation for invasiveness, we found that the proportion of Gram‐negative bacteria for whole body samples was higher for native ants from temperate habitats (*N* = 20) than for invasive ants from temperate habitats (*N* = 9), invasive ants from tropical habitats (*N* = 13), or native ants from tropical habitats (*N* = 51) (Invasiveness: Chisq = 5889.2, *p* < 0.001; Climate: Chisq = 2515.8, *p* < 0.001; Table 1; Figure 3B). In this additive model, despite species‐level differences and study effects explaining a similar and important amount of variance (Table 1), predictors were still significant. Also, we found a strong phylogenetic signal in the proportion of Gram‐negative bacteria for whole body bacterial communities among ant hosts (Figure 4C). Altogether, these findings suggest that for whole body bacterial communities, the external conditions may play a more significant role in shaping these communities than species identity and phylogenetic history (Figure 4C). Among the higher proportions of GN bacteria in temperate ants' whole body bacterial communities, the most abundant taxa belonged to the family *Acetobacteraceae*, the order *Opitutales*, and the genera *Wolbachia* and *Blochmannia*. In native ants, GN taxa in whole body bacterial communities mainly belonged to the family *Enterobacteriaceae* and the genera *Blochmannia*, *Gluconobacter*, and *Wolbachia* (Table S2).

The patterns observed for addictive models were partially recovered when analyzing microscales (arboreal: *N* = 43 vs. ground: *N* = 120) and macroscale (temperate: *N* = 55 vs. tropical: *N* = 138) separately (models with single predictors). The proportion of Gram‐negative bacteria in ant gut bacterial communities presented no significant difference when modeling in response only to microhabitat or climate (Table 1; Figures S3 and S4), suggesting the addictive effects of these predictors do play a role in shaping these communities. Contrarily, whole body bacterial communities keep showing significant differences between climate and invasiveness when running models with single predictors, with ants from temperate habitats presenting higher proportions of Gram‐negative bacteria as well as native ants (Table 1).

Finally, we found no significant influence of sequencing platforms or the use of negative controls on the proportion of gram‐negative bacteria in bacterial communities (Supporting Information 1, Table S3).

## 4 Discussion

Our findings reveal that ants from more climatically variable environments, such as temperate climates and arboreal microhabitats, host a higher proportion of Gram‐negative (GN) bacteria in both their whole body and gut bacterial communities. Also, we found a strong phylogenetic signal in the proportion of Gram‐negative bacteria for whole body bacterial communities among ant hosts This pattern suggests that environmental variability may drive microbial community composition, potentially favoring GN bacteria, which might enhance host resistance and response to environmental conditions under both macro (tropical vs. temperate zones) and microscales (arboreal vs. ground habitats). These results align with the hypothesis that microbial communities contribute to host adaptation in response to ecological variation, particularly in less stable habitats where physiological plasticity is advantageous (temperate zones and canopies). By linking the GN proportion in ants' bacterial communities to more variable habitats, our study provides insights into the ecological and evolutionary dynamics of insect‐microbe interactions.

Partially supporting our initial hypothesis, we found that ants from temperate regions harbor a greater proportion of GN bacteria for whole body, but not for gut bacterial communities. Climate is a well‐established driver of external host‐associated bacterial communities, while internal bacterial communities are shaped by immune complexity, trophic interactions, and climate (Woodhams et al. 2020). Insects' bacterial communities have been shown to facilitate adaptation to daily and seasonal temperature fluctuations, enhancing host resistance to abiotic stress (Ferguson et al. 2018; Ren et al. 2023). However, our study specifically highlights the dominance of GN bacteria in ants' bacterial communities as a potential adaptation to climate variability, an aspect not explicitly addressed in previous works. The most abundant GN taxa found in ants' whole body bacterial communities lack direct evidence of thermal resistance under climate variability, and such information is currently unavailable. Nevertheless, GN bacteria more broadly may provide physiological advantages in fluctuating environments by synthesizing stress‐response proteins, such as CspA, DnaK, DnaJ, and groEl, which help the cell cope with temperature shocks (Ramos et al. 2001). Given the increasing variability of climate patterns, microbial communities may play a critical role in buffering insects against environmental instability. Future research should explore the mechanisms underlying these microbial responses, particularly in the context of climate change (Iltis et al. 2022), and explore how GN bacteria associated with insect hosts contribute to host resilience under temperature fluctuations.

Similarly, our findings indicate that arboreal ants host a higher proportion of GN bacteria, potentially enhancing their resistance in these habitats. Gram‐negative lineages such as *Acetobacteraceae*, *Opitutales*, and *Burkholderiales* were among the most abundant taxa associated with arboreal ants in our dataset. While direct evidence linking these specific taxa to thermal resistance is limited, *Burkholderia* species have been found to accumulate polyhydroxyalkanoates (PHAs), which confer resistance to environmental stresses, including high temperature (Kim et al. 2017). Arboreal ants are known for their greater thermal tolerance compared to ground‐dwelling species (Leahy et al. 2021; Leong et al. 2020; Leponce et al. 2021). Despite several adaptations to the variable and extreme weather conditions in the canopy, such as large nests and numerous colonies distributed in mosaics (Ribeiro et al. 2013; Soares et al. 2022), previous studies have shown that insect‐associated bacteria mediate tolerance to environmental stressors (Ferguson et al. 2018; Gruntenko et al. 2017; Lemoine et al. 2020), and may reshape the composition of foliage bacterial communities where ants forage intensively (Bitar et al. 2021, 2024). Our study is the first to emphasize the specific role of GN bacteria in facilitating resistance to climatic variation in arboreal ants. These results highlight the importance of associated bacteria in host adaptation to microhabitat‐specific stressors, underscoring the need for further investigation into their functional roles.

We acknowledge that beyond environmental conditions, other factors, such as diet, can also influence ants' bacterial communities. Arboreal habitats influence foraging behavior and trophic interactions (Carroll and Janzen 1973), and our data show that herbivorous ants, predominantly arboreal, harbor a higher proportion of GN bacteria than predatory ants, which are primarily ground‐dwelling. Mutualistic microbes often support dietary specialization, influencing nutrient assimilation and host success. For example, *Blochmannia* bacteria in carpenter ants supply essential amino acids (Feldhaar et al. 2007), *Cephalotes* ants rely on microbial symbionts to optimize phloem sap digestion (Pringle and Moreau 2017), and Attini ants cultivate fungi with the support of gut‐associated Mollicutes, Proteobacteria, and Actinobacteria (Sapountzis et al. 2019). In contrast, ground‐dwelling ants, which are predominantly fungivores and predators, exhibit bacterial communities dominated by Mollicutes and Gram‐positive (GP) bacteria, respectively. These results indicate that while climate was our primary focus, diet also appears to be an important factor shaping ant bacterial communities and influencing the presence of Gram‐negative bacteria. Given the number of samples, we were not able to analyze the individual contribution of diet and habitat (Figure S1), which still is an unanswered question that requires further investigation in future studies.

Importantly, this meta‐analysis aimed to examine bacterial communities at the broadest level by quantifying the proportions of GN bacteria associated with ants, rather than focusing on specific taxa. This approach allows for a more general understanding of how fundamental microbial cell structures may relate to environmental tolerance and host adaptation. GN bacteria, characterized by their double‐membrane architecture and diverse metabolic capabilities, may be inherently more suited to fluctuating conditions. However, the ecological consequences of this structural difference remain underexplored in symbiotic systems. An important limitation of our approach is that the categories Gram‐negative and Gram‐positive are not phylogenetically monophyletic, but rather phenotypic groupings that encompass evolutionarily distant lineages. For example, Proteobacteria and Bacteroidetes are both Gram‐negative, whereas Firmicutes and Actinobacteria are Gram‐positive. Some bacteria, including *Mycobacterium*, do not fit neatly into this dichotomy due to their acid‐fast cell wall structure. Although such taxa are not abundant in ant‐associated microbiota, they were classified as Gram‐positive in our analysis, which may slightly oversimplify their cell wall characteristics. Despite this lack of phylogenetic coherence, the GN/GP distinction represents a meaningful ecological trait, as differences in cell wall architecture are linked to bacterial resistance to environmental stressors and to host interactions. Our results should therefore be interpreted as reflecting functional differences between these two broad groups, while future research may benefit from a more lineage‐specific or phylogenetically explicit perspective.

In conclusion, our findings suggest that the composition of ant‐associated bacterial communities is associated with climatic variability across both macro (climatic zone) and micro (habitat structure) scales. Ants in less stable environments tend to harbor bacterial communities dominated by GN bacteria, pointing to a potential role for these microbes in mediating host responses to climatic fluctuations. Moving forward, experimental studies are needed to test the functional consequences of GN dominance for host physiology under thermal fluctuations. Approaches could include controlled manipulation of bacteria, transcriptomic analyses of host stress responses, and experimental evolution under variable climate regimes. We also advocate for more systematic efforts to classify and report the Gram identity of microbial taxa in studies of insect‐associated bacterial communities. As climate change continues to alter the stability of terrestrial ecosystems, understanding how microbial communities contribute to insect resilience will be essential for predicting species persistence and ecosystem function.

## Author Contributions

**M. R. Bitar:** conceptualization (equal), data curation (equal), formal analysis (equal), investigation (equal), methodology (equal), visualization (equal), writing – original draft (equal), writing – review and editing (equal). **M. Azevedo‐Silva:** data curation (equal), formal analysis (equal), investigation (equal), methodology (equal), resources (equal), validation (equal), visualization (equal), writing – review and editing (equal). **P. S. Oliveira:** funding acquisition (equal), supervision (equal), validation (equal), writing – review and editing (equal). **G. Q. Romero:** conceptualization (equal), data curation (equal), formal analysis (equal), investigation (equal), methodology (equal), resources (equal), supervision (equal), validation (equal), writing – review and editing (equal). **S. P. Ribeiro:** conceptualization (equal), data curation (equal), formal analysis (equal), investigation (equal), methodology (equal), project administration (equal), resources (equal), supervision (equal), validation (equal), writing – review and editing (equal).

## Conflicts of Interest

The authors declare no conflicts of interest.

## Supporting information


**Appendix S1:** Supporting Information.

**Appendix S2:** Supporting Information.

**Appendix S3:** Supporting Information.

**Appendix S4:** Supporting Information.

## Data Availability

The datasets supporting the conclusions of this article are included as Supporting Information (Appendices S2 and S4).

## References

[ece372425-bib-0001] Atanaskovic, I. , C. Sharp , C. Press , R. Kaminska , and C. Kleanthous . 2022. “Bacterial Competition Systems Share a Domain Required for Inner Membrane Transport of the Bacteriocin Pyocin G From *Pseudomonas aeruginosa* .” MBio 13: e0339621. 10.1128/mbio.03396-21. 35343790 PMC9040868

[ece372425-bib-0002] Balhuizen, M. D. , A. van Dijk , J. W. A. Jansen , C. H. A. van de Lest , E. J. A. Veldhuizen , and H. P. Haagsman . 2021. “Outer Membrane Vesicles Protect Gram‐Negative Bacteria Against Host Defense Peptides.” mSphere 6: e0052321. 10.1128/msphere.00523-21. 34232080 PMC8386409

[ece372425-bib-0003] Bitar, M. R. , V. D. Pinto , L. M. Moreira , and S. P. Ribeiro . 2021. “Gram‐Negative Bacteria Associated With a Dominant Arboreal Ant Species Outcompete Phyllosphere‐Associated Bacteria Species in a Tropical Canopy.” Oecologia 195: 959–970. 10.1007/s00442-021-04878-y. 33630170

[ece372425-bib-0004] Bitar, M. R. , L. M. R. Tomé , F. V. Costa , et al. 2024. “Bacterial Communities Associated With a Polydomous Arboreal Ant: Inter‐Nest Variation and Interaction With the Phyllosphere of a Tropical Tree.” Myrmecological News 34: 119–127. 10.25849/myrmecol.news_034:119.

[ece372425-bib-0005] Borowiec, M. L. , Y. M. Zhang , K. Neves , et al. 2025. “Evaluating UCE Data Adequacy and Integrating Uncertainty in a Comprehensive Phylogeny of Ants.” Systematic Biology 74: syaf001. 10.1093/sysbio/syaf001. PMC12700001 39774667

[ece372425-bib-0006] Boursaux‐Eude, C. , and R. Gross . 2000. “New Insights Into Symbiotic Associations Between Ants and Bacteria.” Research in Microbiology 151: 513–519. 10.1016/S0923-2508(00)00221-7. 11037129

[ece372425-bib-0007] Brooks, M. E. , K. Kristensen , K. J. van Benthem , et al. 2017. “Modeling Zero‐Inflated Count Data With glmmTMB.” 10.1101/132753.

[ece372425-bib-0008] Cao, J. , C. Wu , K. Wang , et al. 2021. “Metagenomic Profiling Reveals Dominance of Gram‐Positive Bacteria in the Gut Microbiome Shifts Associated With Immunoglobulin A Vasculitis (Henoch–Schönlein Purpura).” Clinical & Translational Immunology 10: e1342. 10.1002/cti2.1342. 34646556 PMC8499602

[ece372425-bib-0009] Carroll, C. R. , and D. H. Janzen . 1973. “Ecology of Foraging by Ants.” Annual Review of Ecology and Systematics 4: 231–257.

[ece372425-bib-0010] Chen, B. , B. S. Teh , C. Sun , et al. 2016. “Biodiversity and Activity of the Gut Microbiota Across the Life History of the Insect Herbivore *Spodoptera littoralis* .” Scientific Reports 6: 29505. 10.1038/srep29505. 27389097 PMC4937375

[ece372425-bib-0011] Coyte, K. Z. , J. Schluter , and K. R. Foster . 2015. “The Ecology of the Microbiome: Networks, Competition, and Stability.” Science 350: 663–666. 10.1126/science.aad2602. 26542567

[ece372425-bib-0012] Deng, Y. , J. Dong , W. Zhang , et al. 2022. “Quantifying the Vertical Microclimate Profile Within a Tropical Seasonal Rainforest, Based on Both Ground‐And Canopy‐Referenced Approaches.” iForest 15: 24–32. 10.3832/ifor3780-014.

[ece372425-bib-0013] Engel, P. , and N. A. Moran . 2013. “The Gut Microbiota of Insects—Diversity in Structure and Function.” FEMS Microbiology Reviews 37: 699–735. 10.1111/1574-6976.12025. 23692388

[ece372425-bib-0014] Fanin, N. , P. Kardol , M. Farrell , M. C. Nilsson , M. J. Gundale , and D. A. Wardle . 2019. “The Ratio of Gram‐Positive to Gram‐Negative Bacterial PLFA Markers as an Indicator of Carbon Availability in Organic Soils.” Soil Biology and Biochemistry 128: 111–114. 10.1016/j.soilbio.2018.10.010.

[ece372425-bib-0015] Feldhaar, H. , J. Straka , M. Krischke , et al. 2007. “Nutritional Upgrading for Omnivorous Carpenter Ants by the Endosymbiont Blochmannia.” BMC Biology 5: 48. 10.1186/1741-7007-5-48. 17971224 PMC2206011

[ece372425-bib-0016] Ferguson, L. V. , P. Dhakal , J. E. Lebenzon , D. E. Heinrichs , C. Bucking , and B. J. Sinclair . 2018. “Seasonal Shifts in the Insect Gut Microbiome Are Concurrent With Changes in Cold Tolerance and Immunity.” Functional Ecology 32: 2357–2368. 10.1111/1365-2435.13153.

[ece372425-bib-0017] Galdiero, S. , A. Falanga , M. Cantisani , et al. 2012. “Microbe‐Host Interactions: Structure and Role of Gram‐Negative Bacterial Porins.” Current Protein and Peptide Science 13: 843–854. 23305369 10.2174/138920312804871120 PMC3706956

[ece372425-bib-0018] Green, E. A. , and J. L. Klassen . 2022. “ *Trachymyrmex septentrionalis* Ant Microbiome Assembly Is Unique to Individual Colonies and Castes.” mSphere 7: e0098921. 10.1128/msphere.00989-21. 35862804 PMC9429924

[ece372425-bib-0019] Gruntenko, N. E. , Y. Y. Ilinsky , N. V. Adonyeva , et al. 2017. “Various Wolbachia Genotypes Differently Influence Host Drosophila Dopamine Metabolism and Survival Under Heat Stress Conditions.” BMC Evolutionary Biology 17: 252. 10.1186/s12862-017-1104-y. 29297293 PMC5751659

[ece372425-bib-0020] Gupta, A. , and S. Nair . 2020. “Dynamics of Insect–Microbiome Interaction Influence Host and Microbial Symbiont.” Frontiers in Microbiology 11: 1357. 10.3389/fmicb.2020.01357. 32676060 PMC7333248

[ece372425-bib-0021] Gupta, R. S. 2011. “Origin of Diderm (Gram‐Negative) Bacteria: Antibiotic Selection Pressure Rather Than Endosymbiosis Likely Led to the Evolution of Bacterial Cells With Two Membranes.” Antonie van Leeuwenhoek 100: 171–182. 10.1007/s10482-011-9616-8. 21717204 PMC3133647

[ece372425-bib-0022] Hammer, T. J. , J. G. Sanders , and N. Fierer . 2019. “Not All Animals Need a Microbiome.” FEMS Microbiology Letters 366: fnz117. 10.1093/femsle/fnz117. 31132110

[ece372425-bib-0023] Hannula, S. E. , F. Zhu , R. Heinen , and T. M. Bezemer . 2019. “Foliar‐Feeding Insects Acquire Microbiomes From the Soil Rather Than the Host Plant.” Nature Communications 10: 1254. 10.1038/s41467-019-09284-w. PMC6425034 30890706

[ece372425-bib-0024] Harvey, J. A. , K. Tougeron , R. Gols , et al. 2023. “Scientists' Warning on Climate Change and Insects.” Ecological Monographs 93: e1553. 10.1002/ecm.1553.

[ece372425-bib-0025] Hölldobler, B. , and E. O. Wilson . 1990. The Ants. Harvard University Press.

[ece372425-bib-0026] Hu, Y. , D. A. Holway , P. Łukasik , et al. 2017. “By Their Own Devices: Invasive Argentine Ants Have Shifted Diet Without Clear Aid From Symbiotic Microbes.” Molecular Ecology 26: 1608–1630. 10.1111/mec.13991. 28026894

[ece372425-bib-0027] Hu, Y. , P. Łukasik , C. S. Moreau , and J. A. Russell . 2014. “Correlates of Gut Community Composition Across an Ant Species ( *Cephalotes varians* ) Elucidate Causes and Consequences of Symbiotic Variability.” Molecular Ecology 23: 1284–1300. 10.1111/mec.12607. 24286170

[ece372425-bib-0028] Iltis, C. , K. Tougeron , T. Hance , P. Louâpre , and V. Foray . 2022. “A Perspective on Insect–Microbe Holobionts Facing Thermal Fluctuations in a Climate‐Change Context.” Environmental Microbiology 24: 18–29. 10.1111/1462-2920.15826. 34713541

[ece372425-bib-0029] Ivens, A. B. F. , A. Gadau , E. T. Kiers , and D. J. C. Kronauer . 2018. “Can Social Partnerships Influence the Microbiome? Insights From Ant Farmers and Their Trophobiont Mutualists.” Molecular Ecology 27: 1898–1914. 10.1111/mec.14506. 29411455 PMC5935579

[ece372425-bib-0030] Kaltenpoth, M. , L. V. Flórez , A. Vigneron , P. Dirksen , and T. Engl . 2025. “Origin and Function of Beneficial Bacterial Symbioses in Insects.” Nature Reviews Microbiology 23: 551–567. 10.1038/s41579-025-01164-z. 40148601

[ece372425-bib-0031] Khakisahneh, S. , X.‐Y. Zhang , Z. Nouri , and D.‐H. Wang . 2020. “Gut Microbiota and Host Thermoregulation in Response to Ambient Temperature Fluctuations.” mSystems 5: e00514‐20. 10.1128/msystems.00514-20. 33082280 PMC7577294

[ece372425-bib-0032] Kim, B. R. , J. Shin , R. B. Guevarra , et al. 2017. “Deciphering Diversity Indices for a Better Understanding of Microbial Communities.” Journal of Microbiology and Biotechnology 27: 2089–2093. 10.4014/jmb.1709.09027. 29032640

[ece372425-bib-0033] Konstantinidis, K. T. , and J. M. Tiedje . 2005. “Genomic Insights That Advance the Species Definition for Prokaryotes.” PNAS 102: 2567–2572. 15701695 10.1073/pnas.0409727102 PMC549018

[ece372425-bib-0034] Lange, C. , S. Boyer , T. M. Bezemer , et al. 2023. “Impact of Intraspecific Variation in Insect Microbiomes on Host Phenotype and Evolution.” ISME Journal 17: 1798–1807. 10.1038/s41396-023-01500-2. 37660231 PMC10579242

[ece372425-bib-0035] Leahy, L. , B. R. Scheffers , A. N. Andersen , B. T. Hirsch , and S. E. Williams . 2021. “Vertical Niche and Elevation Range Size in Tropical Ants: Implications for Climate Resilience.” Diversity and Distributions 27: 485–496. 10.1111/ddi.13210.

[ece372425-bib-0036] Lemoine, M. M. , T. Engl , and M. Kaltenpoth . 2020. “Microbial Symbionts Expanding or Constraining Abiotic Niche Space in Insects.” Current Opinion in Insect Science 39: 14–20. 10.1016/j.cois.2020.01.003. 32086000

[ece372425-bib-0037] Leong, C.‐M. , T. P. N. Tsang , and B. Guénard . 2020. “Critical Thermal Maximum Measurements and Its Biological Relevance: The Case of Ants.” 10.1101/2020.12.09.417410.

[ece372425-bib-0038] Leponce, M. , A. Dejean , O. Mottl , and P. Klimes . 2021. “Rapid Assessment of the Three‐Dimensional Distribution of Dominant Arboreal Ants in Tropical Forests.” Insect Conservation and Diversity 14: 426–438. 10.1111/icad.12486.

[ece372425-bib-0039] Lisovski, S. , M. Ramenofsky , and J. C. Wingfield . 2017. “Defining the Degree of Seasonality and Its Significance for Future Research.” In Integrative and Comparative Biology, vol. 57, 934–942. Oxford University Press. 10.1093/icb/icx040. 28662570

[ece372425-bib-0040] Liukkonen, M. , J. Muriel , J. Martínez‐Padilla , et al. 2024. “Seasonal and Environmental Factors Contribute to the Variation in the Gut Microbiome: A Large‐Scale Study of a Small Bird.” Journal of Animal Ecology 93: 1475–1492. 10.1111/1365-2656.14153. 39041321

[ece372425-bib-0041] Lucas, J. , B. Bill , B. Stevenson , and M. Kaspari . 2017. “The Microbiome of the Ant‐Built Home: The Microbial Communities of a Tropical Arboreal Ant and Its Nest.” Ecosphere 8: e01639. 10.1002/ecs2.1639.

[ece372425-bib-0042] Lucas, J. M. , A. A. Madden , C. A. Penick , et al. 2019. “Azteca Ants Maintain Unique Microbiomes Across Functionally Distinct Nest Chambers.” Proceedings of the Royal Society B: Biological Sciences 286: 20191026. 10.1098/rspb.2019.1026. PMC6710589 31387509

[ece372425-bib-0043] Magoga, G. , M. Brunetti , L. Kajtoch , A. Spada , and M. Montagna . 2023. “Biotic and Abiotic Factors Affecting the Microbiota of Chrysomelidae Inhabiting Wetland Vegetation.” Hydrobiologia 850: 3797–3812. 10.1007/s10750-022-05082-6.

[ece372425-bib-0044] Marsico, T. D. , J. W. Burt , E. K. Espeland , et al. 2010. “Underutilized Resources for Studying the Evolution of Invasive Species During Their Introduction, Establishment, and Lag Phases.” Evolutionary Applications 3: 203–219. 10.1111/j.1752-4571.2009.00101.x. 25567920 PMC3352478

[ece372425-bib-0045] McMunn, M. S. , A. I. Hudson , A. T. Zemenick , et al. 2022. “Thermal Sensitivity and Seasonal Change in the Gut Microbiome of a Desert Ant, *Cephalotes rohweri* .” FEMS Microbiology Ecology 98: fiac062. 10.1093/femsec/fiac062. 35641145

[ece372425-bib-0046] Mondal, S. , J. Somani , S. Roy , A. Babu , and A. K. Pandey . 2023. “Insect Microbial Symbionts: Ecology, Interactions, and Biological Significance.” Microorganisms 11: 2665. 10.3390/microorganisms11112665. 38004678 PMC10672782

[ece372425-bib-0047] Montllor, C. B. , A. Maxmen , and A. H. Purcell . 2002. “Facultative Bacterial Endosymbionts Benefit Pea Aphids *Acyrthosiphon pisum* Under Heat Stress.” Ecological Entomology 27: 189–195. 10.1046/j.1365-2311.2002.00393.x.

[ece372425-bib-0048] Morales, M. 2022. Package ‘sciplot’. CRAN web host. https://www.rdocumentation.org/packages/sciplot.

[ece372425-bib-0049] Moran, N. A. , J. A. Russell , R. Koga , and T. Fukatsu . 2005. “Evolutionary Relationships of Three New Species of Enterobacteriaceae Living as Symbionts of Aphids and Other Insects.” Applied and Environmental Microbiology 71: 3302–3310. 10.1128/AEM.71.6.3302-3310.2005. 15933033 PMC1151865

[ece372425-bib-0050] Moreau, C. S. 2020. “Symbioses Among Ants and Microbes.” Current Opinion in Insect Science 39: 1–5. 10.1016/j.cois.2020.01.002. 32078984

[ece372425-bib-0051] Nikaido, H. 2003. “Molecular Basis of Bacterial Outer Membrane Permeability Revisited.” Microbiology and Molecular Biology Reviews 67: 593–656. 10.1128/mmbr.67.4.593-656.2003. 14665678 PMC309051

[ece372425-bib-0052] Page, M. J. , J. E. McKenzie , P. M. Bossuyt , et al. 2021. “The PRISMA 2020 Statement: An Updated Guideline for Reporting Systematic Reviews.” BMJ 372: n71. 10.1136/bmj.n71. 33782057 PMC8005924

[ece372425-bib-0053] Paradis, E. , and K. Schliep . 2019. “Ape 5.0: An Environment for Modern Phylogenetics and Evolutionary Analyses in R.” Bioinformatics 35: 526–528. 10.1093/bioinformatics/bty633. 30016406

[ece372425-bib-0054] Pringle, E. G. , and C. S. Moreau . 2017. “Community Analysis of Microbial Sharing and Specialization in a Costa Rican Ant—Plant—Hemipteran Symbiosis.” Proceedings of the Royal Society B: Biological Sciences 284: 20162770. 10.1098/rspb.2016.2770. PMC5360929 28298351

[ece372425-bib-0055] R Core Team . 2021. R: A Language and Environment for Statistical Computing.

[ece372425-bib-0056] Ramos, J. L. , M. T. Gallegos , S. Marqués , M. I. Ramos‐González , M. Espinosa‐Urgel , and A. Segura . 2001. “Responses of Gram‐Negative Bacteria to Certain Environmental Stressors.” Current Opinion in Microbiology 4, no. 2: 166–171. 10.1016/S1369-5274(00)00183-1. 11282472

[ece372425-bib-0057] Raza, M. F. , Y. Wang , Z. Cai , et al. 2020. “Gut Microbiota Promotes Host Resistance to Low‐Temperature Stress by Stimulating Its Arginine and Proline Metabolism Pathway in Adult *Bactrocera dorsalis* .” PLoS Pathogens 16: e1008441. 10.1371/journal.ppat.1008441. 32294136 PMC7185725

[ece372425-bib-0058] Ren, L. , X. Zhang , F. Yang , et al. 2023. “Effects of Heat Tolerance on the Gut Microbiota of Sarcophaga Peregrina (Diptera: Sarcophagidae) and Impacts on the Life History Traits.” Parasites & Vectors 16: 364. 10.1186/s13071-023-05973-0. 37848940 PMC10580603

[ece372425-bib-0059] Revell, L. J. 2024. “Phytools 2.0: An Updated R Ecosystem for Phylogenetic Comparative Methods (and Other Things).” PeerJ 12: e16505. 10.7717/peerj.16505. 38192598 PMC10773453

[ece372425-bib-0060] Ribeiro, S. P. , N. B. E. Santo , J. H. C. Delabie , and J. D. Majer . 2013. “Competition, Resources and the Ant (Hymenoptera: Formicidae) Mosaic: A Comparison of Upper and Lower Canopy.” Myrmecological News 18: 113–120.

[ece372425-bib-0061] Rocha, F. P. , M. U. V. Ronque , M. L. Lyra , M. Bacci , and P. S. Oliveira . 2023. “Habitat and Host Species Drive the Structure of Bacterial Communities of Two Neotropical Trap‐Jaw Odontomachus Ants: Habitat and Host Species Drive the Structure of Bacterial Communities of Two Neotropical Trap‐Jaw Odontomachus Ants.” Microbial Ecology 86: 699–712. 10.1007/s00248-022-02064-y. 35802173

[ece372425-bib-0062] Ronque, M. U. V. , M. L. Lyra , G. H. Migliorini , M. Bacci , and P. S. Oliveira . 2020. “Symbiotic Bacterial Communities in Rainforest Fungus‐Farming Ants: Evidence for Species and Colony Specificity.” Scientific Reports 10: 10172. 10.1038/s41598-020-66772-6. 32576863 PMC7311517

[ece372425-bib-0063] Russell, J. A. 2017. “Hotspots for Symbiosis: Function, Evolution, and Specificity of Ant‐Microbe Associations From Trunk to Tips of the Ant Phylogeny (Hymenoptera: Formicidae).” Myrmecological News 24: 43–69.

[ece372425-bib-0064] Sapountzis, P. , D. R. Nash , M. Schiøtt , and J. J. Boomsma . 2019. “The Evolution of Abdominal Microbiomes in Fungus‐Growing Ants.” Molecular Ecology 28: 879–899. 10.1111/mec.14931. 30411820 PMC6446810

[ece372425-bib-0065] Scheffers, B. R. , and S. E. Williams . 2018. “Tropical Mountain Passes Are Out of Reach—But Not for Arboreal Species.” Frontiers in Ecology and the Environment 16: 101–108. 10.1002/fee.1764.

[ece372425-bib-0066] Schneider, C. A. , W. S. Rasband , and K. W. Eliceiri . 2012. “NIH Image to ImageJ: 25 Years of Image Analysis.” Nature Methods 9, no. 7: 671–675. 10.1038/nmeth.2089. 22930834 PMC5554542

[ece372425-bib-0067] Schwechheimer, C. , C. J. Sullivan , and M. J. Kuehn . 2013. “Envelope Control of Outer Membrane Vesicle Production in Gram‐Negative Bacteria.” Biochemistry 52: 3031–3040. 10.1021/bi400164t. 23521754 PMC3731998

[ece372425-bib-0068] Segers, F. H. I. D. , M. Kaltenpoth , and S. Foitzik . 2019. “Abdominal Microbial Communities in Ants Depend on Colony Membership Rather Than Caste and Are Linked to Colony Productivity.” Ecology and Evolution 9: 13450–13467. 10.1002/ece3.5801. 31871657 PMC6912891

[ece372425-bib-0069] Silhavy, T. J. , D. Kahne , and S. Walker . 2010. “The Bacterial Cell Envelope.” Cold Spring Harbor Perspectives in Biology 2: a000414. 10.1101/cshperspect.a000414. 20452953 PMC2857177

[ece372425-bib-0070] Soares, G. R. , G. M. Lourenço , F. V. Costa , et al. 2022. “Territory and Trophic Cascading Effects of the Ant *Azteca chartifex* (Hymenoptera: Formicidae) in a Tropical Canopy.” Myrmecological News 32: 103–113. 10.25849/myrmecol.news_032:103.

[ece372425-bib-0071] Vinod, N. , M. Slot , I. R. McGregor , et al. 2023. “Thermal Sensitivity Across Forest Vertical Profiles: Patterns, Mechanisms, and Ecological Implications.” New Phytologist 237: 22–47. 10.1111/nph.18539. 36239086

[ece372425-bib-0072] Wang, Z. , Y. Wu , X. Li , X. Ji , and W. Liu . 2024. “The Gut Microbiota Facilitate Their Host Tolerance to Extreme Temperatures.” BMC Microbiology 24: 131. 10.1186/s12866-024-03277-6. 38643098 PMC11031955

[ece372425-bib-0073] Wickham, H. 2009. ggplot2. Springer New York. 10.1007/978-0-387-98141-3.

[ece372425-bib-0074] Woodhams, D. C. , M. C. Bletz , C. G. Becker , et al. 2020. “Host‐Associated Microbiomes Are Predicted by Immune System Complexity and Climate.” Genome Biology 21: 23. 10.1186/s13059-019-1908-8. 32014020 PMC6996194

[ece372425-bib-0075] Yu, G. , D. K. Smith , H. Zhu , Y. Guan , and T. T. Y. Lam . 2017. “Ggtree: An R Package for Visualization and Annotation of Phylogenetic Trees With Their Covariates and Other Associated Data.” Methods in Ecology and Evolution 8: 28–36. 10.1111/2041-210X.12628.

[ece372425-bib-0076] Zhang, Y. , T. Cai , M. Yuan , et al. 2023. “Microbiome Variation Correlates With the Insecticide Susceptibility in Different Geographic Strains of a Significant Agricultural Pest, *Nilaparvata lugens* .” npj Biofilms and Microbiomes 9: 2. 10.1038/s41522-023-00369-5. 36635299 PMC9837087

[ece372425-bib-0077] Zhang, Y. , S. Zhang , and L. Xu . 2023. “The Pivotal Roles of Gut Microbiota in Insect Plant Interactions for Sustainable Pest Management.” npj Biofilms and Microbiomes 9: 66. 10.1038/s41522-023-00435-y. 37735530 PMC10514296

