# Supplementary material for: Gram‐Negative Bacteria Across Spatial Scales: A Meta‐Analysis of Ant‐Associated Bacterial Communities Under Distinct Environmental Conditions
Source: Ecol Evol. 2025 Oct 30;15(11):e72425. doi: 10.1002/ece3.72425 (PMC12575185; doi:10.1002/ece3.72425)
Supplement: Supplementary file 1 — Appendix S1: Supporting Information. [file ECE3-15-e72425-s004.docx]

**Prevalence of Gram-negative bacteria across spatial scales: A meta-analysis of ant-associated bacterial communities in changing environments**

Bitar MR^1,2^, Azevedo-Silva M³, Oliveira PS², Romero GQ^2^, Ribeiro SP^1^

^1^Laboratório de Ecologia do Adoecimento & Florestas NUPEB/ICEB, Universidade Federal de Ouro Preto, Ouro Preto, Minas Gerais, Brazil

^2^Departamento de Biologia Animal, Universidade Estadual de Campinas (UNICAMP), Campinas, São Paulo, CEP 13083-862, Brazil

³Department of Ecology and Evolutionary Biology, University of Michigan, Ann Arbor, Michigan, USA

**Supplementary Information**


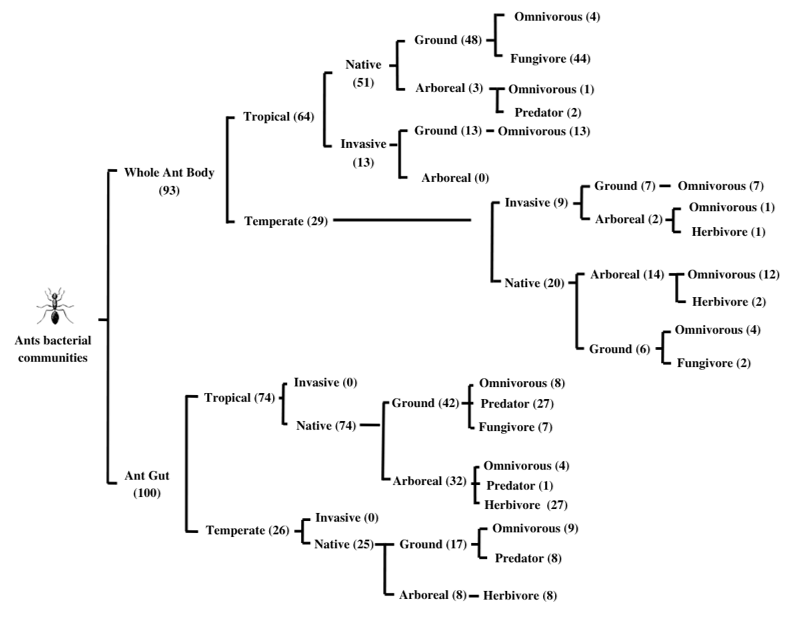


Fig S1 - Scheme representing the hierarchical organization of predictors tested and the number of outputs in each category in parentheses.

**
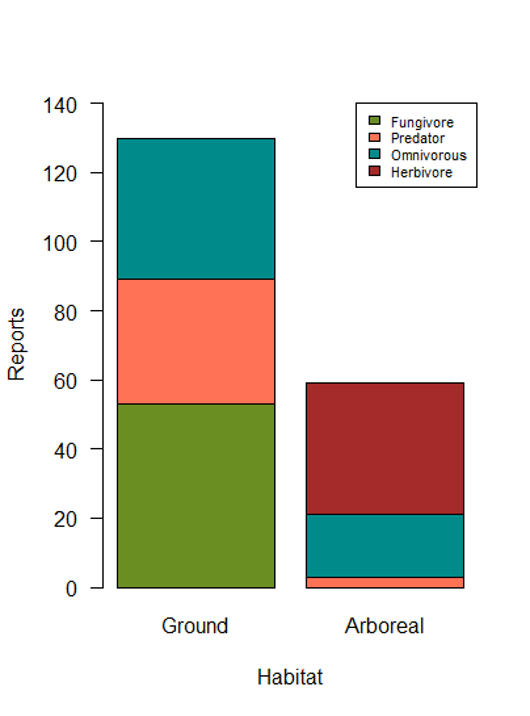
**

Fig S2 – Barplot showing the number of reports analyzing ants’ bacterial communities from Ground and Arboreal habitats. Different colors represent the ants’ diets corresponding to their habitats.

**
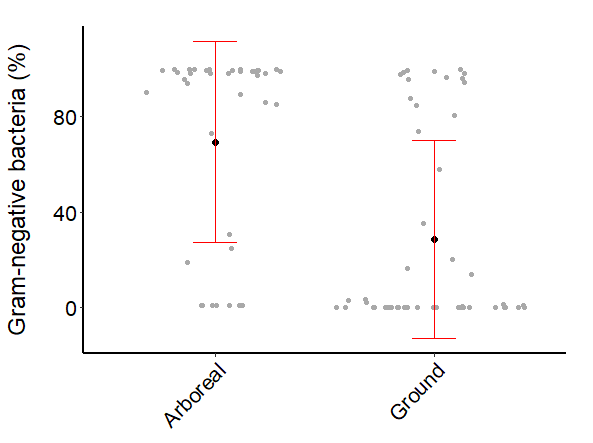
**

Fig S3 - Comparison of the proportion of Gram-negative bacteria in ant’s gut bacterial communities between microscale environments (arboreal *versus* ground habitats).


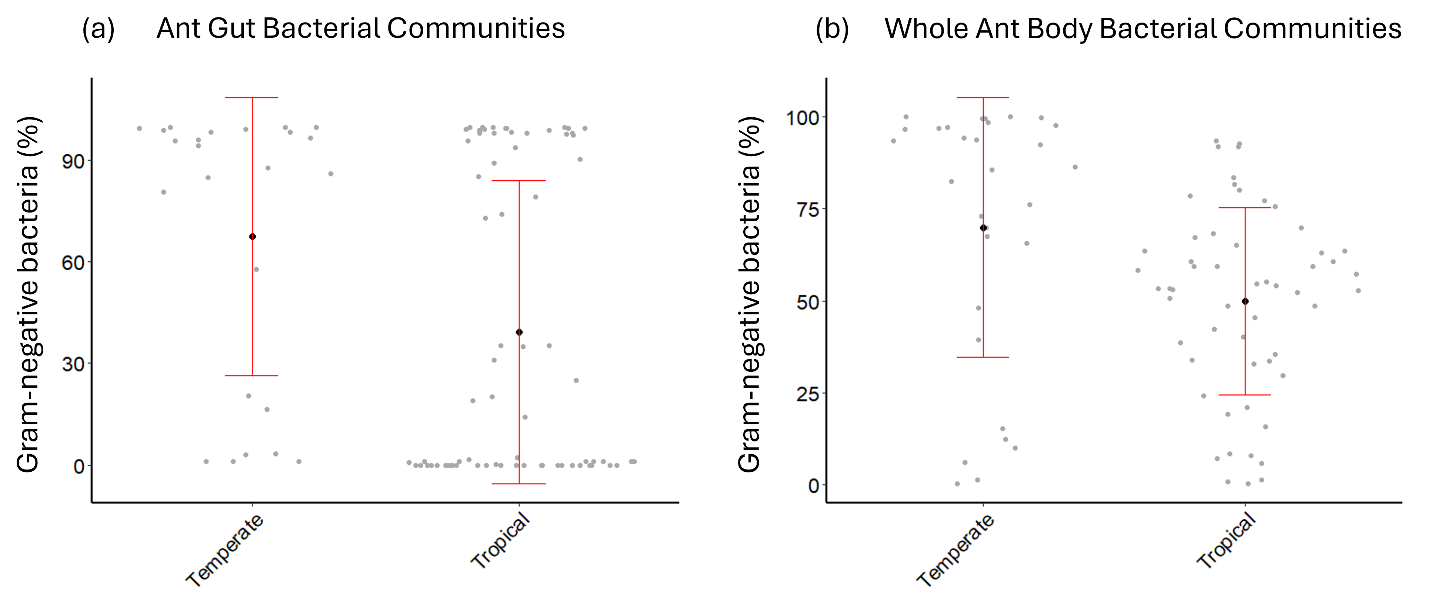


Fig S4 - Comparison of the proportion of Gram-negative bacteria in bacterial communities of ant’s (a) gut and (b) whole body between macroscale environments (temperate *versus* tropical regions).
