# Supplementary material for: Gram‐Negative Bacteria Across Spatial Scales: A Meta‐Analysis of Ant‐Associated Bacterial Communities Under Distinct Environmental Conditions
Source: Ecol Evol. 2025 Oct 30;15(11):e72425. doi: 10.1002/ece3.72425 (PMC12575185; doi:10.1002/ece3.72425)
Supplement: Supplementary file 3 — Appendix S3: Supporting Information. [file ECE3-15-e72425-s001.docx]

**Prevalence of Gram-negative bacteria across spatial scales: A meta-analysis of ant-associated bacterial communities in changing environments**

Bitar MR^1,2^, Azevedo-Silva M³, Oliveira PS², Romero GQ^2^, Ribeiro SP^1^

^1^Laboratório de Ecologia do Adoecimento & Florestas NUPEB/ICEB, Universidade Federal de Ouro Preto, Ouro Preto, Minas Gerais, Brazil

^2^Departamento de Biologia Animal, Universidade Estadual de Campinas (UNICAMP), Campinas, São Paulo, CEP 13083-862, Brazil

³Department of Ecology and Evolutionary Biology, University of Michigan, Ann Arbor, Michigan, USA

| **Table S1.** Test of platform sequencing effects on Gram-negative bacteria proportion.   \| Effect \| Estimate \| Standard Error \| Z-value \| P-value \| \| --- \| --- \| --- \| --- \| --- \| \| Intercept \| -0.6700 \| 0.8271 \| -0.810 \| 0.418 \| \| Plataform: Illumina Hiseq \| 0.8634 \| 1.8494 \| 0.467 \| 0.641 \| \| Plataform: Illumina Miseq \| 1.1986 \| 1.0368 \| 1.156 \| 0.248 \| \| Plataform: Ion Torrent PGM \| -1.3267 \| 1.8501 \| -0.717 \| 0.473 \| |
| --- | --- | --- | --- | --- | --- | --- | --- | --- | --- | --- | --- | --- | --- | --- | --- | --- | --- | --- | --- | --- | --- | --- | --- | --- | --- |

**Table S2.** Test of decontamination analysis effects on Gram-negative bacteria proportion.

| Effect | Estimate | Standard Error | Z-value | P-value |
| --- | --- | --- | --- | --- |
| Intercept | -0.001607 | 0.549601 | -0.003 | 0.998 |
| Decontamination: Yes | -0.149830 | 1.079550 | -0.139 | 0.890 |
|  |  |  |  |  |

**Table S3.** Analysis of variance (ANOVA) results conducted for each model to evaluate the influence of sequencing platforms and use of negative controls on the proportion of gram-negative bacteria in bacterial communities. Chisq = Chi-squared statistic; Df = degrees of freedom; P = p-value. Random effects variance and standard deviation (sd) of each GLMM are also shown.

| **Predictors** | **Chisq** | **Df** | **P** | **Random effects** | **Variance ± sd (random effect)** |
| --- | --- | --- | --- | --- | --- |
| **Sequencing strategy** | 2.07 | 4 | 0.723 | Study | 7.44 ± 2.73 |
|  |  |  |  | Species | 11.43 ± 3.38 |
| **Decontamination** | 0.03 | 1 | 0.869 | Study | 7.44 ± 2.73 |
|  |  |  |  | Species | 11.43 ± 3.38 |
