# Supplementary material for: Gram‐Negative Bacteria Across Spatial Scales: A Meta‐Analysis of Ant‐Associated Bacterial Communities Under Distinct Environmental Conditions
Source: Ecol Evol. 2025 Oct 30;15(11):e72425. doi: 10.1002/ece3.72425 (PMC12575185; doi:10.1002/ece3.72425)
Supplement: Supplementary file 4 — Appendix S4: Supporting Information. [file ECE3-15-e72425-s003.docx]

**Prevalence of Gram-negative bacteria across spatial scales: A meta-analysis of ant-associated bacterial communities in changing environments**

Bitar MR^1,2^, Azevedo-Silva M³, Oliveira PS², Romero GQ^2^, Ribeiro SP^1^

^1^Laboratório de Ecologia do Adoecimento & Florestas NUPEB/ICEB, Universidade Federal de Ouro Preto, Ouro Preto, Minas Gerais, Brazil

^2^Departamento de Biologia Animal, Universidade Estadual de Campinas (UNICAMP), Campinas, São Paulo, CEP 13083-862, Brazil

³Department of Ecology and Evolutionary Biology, University of Michigan, Ann Arbor, Michigan, USA

**Meta-analysis references**

1. Ashigar MA, Ab Majid AH. 2021 16S rDNA metabarcoding of the bacterial community associated with workers of Pheidole rugaticeps Emery (Hymenoptera: Formicidae). *J Asia Pac Entomol* 24, 176–183. (doi:10.1016/j.aspen.2020.12.003)

2. Brown BP, Wernegreen JJ. 2016 Deep divergence and rapid evolutionary rates in gut-associated Acetobacteraceae of ants. *BMC Microbiol* 16. (doi:10.1186/s12866-016-0721-8)

3. Chua KO, Song SL, Yong H Sen, See-Too WS, Yin WF, Chan KG. 2018 Microbial Community Composition Reveals Spatial Variation and Distinctive Core Microbiome of the Weaver Ant Oecophylla smaragdina in Malaysia. *Sci Rep* 8. (doi:10.1038/s41598-018-29159-2)

4. Cooling M, Gruber MAM, Hoffmann BD, Sébastien A, Lester PJ. 2017 A metatranscriptomic survey of the invasive yellow crazy ant, Anoplolepis gracilipes, identifies several potential viral and bacterial pathogens and mutualists. *Insectes Soc* 64, 197–207. (doi:10.1007/s00040-016-0531-x)

5. González-Escobar JL, Grajales-Lagunes A, Smoliński A, Chagolla-López A, De Léon-Rodríguez A, Barba de la Rosa AP. 2018 Microbiota of edible Liometopum apiculatum ant larvae reveals potential functions related to their nutritional value. *Food Research International* 109, 497–505. (doi:10.1016/j.foodres.2018.04.049)

6. Arcila Hernández LM, Sanders JG, Miller GA, Ravenscraft A, Frederickson ME. 2017 Ant–plant mutualism: a dietary by-product of a tropical ant’s macronutrient requirements. *Ecology* 98, 3141–3151. (doi:10.1002/ecy.2036)

7. Hosmath KS, Timmappa SC. 2019 Comparison between the microbial diversity in carpenter ant (Camponotus) gut and weaver ant (Oecophylla) gut. *J Pure Appl Microbiol* 13, 24212436. (doi:10.22207/JPAM.13.4.58)

8. Hu Y, Łukasik P, Moreau CS, Russell JA. 2014 Correlates of gut community composition across an ant species (Cephalotes varians) elucidate causes and consequences of symbiotic variability. *Mol Ecol* 23, 1284–1300. (doi:10.1111/mec.12607)

9. Hu Y *et al.* 2017 By their own devices: invasive Argentine ants have shifted diet without clear aid from symbiotic microbes. *Mol Ecol* 26, 1608–1630. (doi:10.1111/mec.13991)

10. Ishak HD, Miller JL, Sen R, Dowd SE, Meyer E, Mueller UG. 2011a Microbiomes of ant castes implicate new microbial roles in the fungus-growing ant Trachymyrmex septentrionalis. *Sci Rep* 1. (doi:10.1038/srep00204)

11. Ishak HD, Plowes R, Sen R, Kellner K, Meyer E, Estrada DA, Dowd SE, Mueller UG. 2011b Bacterial Diversity in Solenopsis invicta and Solenopsis geminata Ant Colonies Characterized by 16S amplicon 454 Pyrosequencing. *Microb Ecol* 61, 821–831. (doi:10.1007/s00248-010-9793-4)

12. Kaczmarczyk-ziemba A, Zagaja M, Wagner GK, Pietrykowska-tudruj E, Staniec B. 2020 First insight into microbiome profiles of myrmecophilous beetles and their host, red wood ant formica polyctena (Hymenoptera: Formicidae)— A case study. *Insects* 11. (doi:10.3390/insects11020134)

13. Kellner K, Ishak HD, Linksvayer TA, Mueller UG. 2015 Bacterial community composition and diversity in an ancestral ant fungus symbiosis. *FEMS Microbiol Ecol* 91. (doi:10.1093/femsec/fiv073)

14. Lester PJ, Sébastien A, Suarez A V., Barbieri RF, Gruber MAM. 2017 Symbiotic bacterial communities in ants are modified by invasion pathway bottlenecks and alter host behavior. *Ecology* 98, 861–874. (doi:10.1002/ecy.1714)

15. Lucas J, Bill B, Stevenson B, Kaspari M. 2017 The microbiome of the ant-built home: The microbial communities of a tropical arboreal ant and its nest. *Ecosphere* 8. (doi:10.1002/ecs2.1639)

16. Łukasik P, Newton JA, Sanders JG, Hu Y, Moreau CS, Kronauer DJC, O’Donnell S, Koga R, Russell JA. 2017 The structured diversity of specialized gut symbionts of the New World army ants. *Mol Ecol* 26, 3808–3825. (doi:10.1111/mec.14140)

17. Moreau CS, Rubin BER. 2017 Diversity and persistence of the gut microbiome of the giant neotropical bullet ant. *Integr Comp Biol*. 57, 682–689. (doi:10.1093/icb/icx037)

18. Pringle EG, Moreau CS. 2017 Community analysis of microbial sharing and specialization in a Costa Rican ant – plant – hemipteran symbiosis. *Proceedings of the Royal Society B: Biological Sciences* 284. (doi:10.1098/rspb.2016.2770)

19. Ramalho MO, Bueno OC, Moreau CS. 2017 Species-specific signatures of the microbiome from Camponotus and Colobopsis ants across developmental stages. *PLoS One* 12. (doi:10.1371/journal.pone.0187461)

20. Ramalho M de O, Martins C, Morini MSC, Bueno OC. 2020 What can the bacterial community of atta sexdens (Linnaeus, 1758) tell us about the habitats in which this ant species evolves? *Insects* 11. (doi:10.3390/insects11060332)

21. Ramalho MO, Duplais C, Orivel J, Dejean A, Gibson JC, Suarez A V., Moreau CS. 2020 Development but not diet alters microbial communities in the Neotropical arboreal trap jaw ant Daceton armigerum: an exploratory study. *Sci Rep* 10. (doi:10.1038/s41598-020-64393-7)

22. Ronque MUV, Lyra ML, Migliorini GH, Bacci M, Oliveira PS. 2020 Symbiotic bacterial communities in rainforest fungus-farming ants: evidence for species and colony specificity. *Sci Rep* 10. (doi:10.1038/s41598-020-66772-6)

23. Di Salvo M, Calcagnile M, Talà A, Tredici SM, Maffei ME, Schönrogge K, Barbero F, Alifano P. 2019 The Microbiome of the Maculinea-Myrmica Host-Parasite Interaction. *Sci Rep* 9. (doi:10.1038/s41598-019-44514-7)

24. Sanders JG, Powell S, Kronauer DJC, Vasconcelos HL, Frederickson ME, Pierce NE. 2014 Stability and phylogenetic correlation in gut microbiota: Lessons from ants and apes. *Mol Ecol* 23, 1268–1283. (doi:10.1111/mec.12611)

25. Vieira AS, Ramalho MO, Martins C, Martins VG, Bueno OC. 2017 Microbial Communities in Different Tissues of Atta sexdens rubropilosa Leaf-cutting Ants. *Curr Microbiol* 74, 1216–1225. (doi:10.1007/s00284-017-1307-x)

26. Zheng Z, Hu X, Xu Y, Wei C, He H. 2021 Bacterial composition and diversity of the digestive tract of odontomachus monticola emery and ectomomyrmex javanus mayr. *Insects* 12, 1–14. (doi:10.3390/insects12020176)

27. Zhukova M, Sapountzis P, Schiøtt M, Boomsma JJ. 2017 Diversity and transmission of gut bacteria in Atta and Acromyrmex leaf-cutting ants during development. *Front Microbiol* 8. (doi:10.3389/fmicb.2017.01942)
